# Social and self-stigma during COVID-19 pandemic: Egyptians’ perspectives

**DOI:** 10.1371/journal.pone.0284582

**Published:** 2023-04-20

**Authors:** Ayat Mahmoud Tawfik, Noha Hayek, Sarah Salah Eldien Mahdy, Noura Mahmoud Elsherbiny, Marwa Rashad Salem, Almass Fathi Hassan Taie

**Affiliations:** 1 Public Health and Community Medicine Department, Faculty of Medicine, Port-Said University, Port-Said, Egypt; 2 Public Health Department, Theodore Bilharz Research Institute, Giza, Egypt; 3 Family Medicine Department, Faculty of Medicine, Suez Canal University, Ismailia, Egypt; 4 Public Health and Community Medicine Department, Faculty of Medicine, Suez Canal University, Ismailia, Egypt; 5 Public Health and Community Medicine Department, Faculty of Medicine, Cairo University, Giza City, Egypt; JSMU: Jinnah Sindh Medical University, PAKISTAN

## Abstract

**Background:**

Social stigma associated with infectious diseases existed throughout the history of pandemics due to fears of contagion and death. This study aims to assess social and self-stigma resulting from COVID-19 infection and other associated factors in Egypt during the pandemic.

**Methods:**

A cross-sectional study was conducted on 533 adult Egyptians via an online questionnaire. The questionnaire included social stigma toward current and recovered COVID-19 patients and the negative self-image of being a COVID-19 patient.

**Results:**

The mean calculated overall COVID-19-related stigma score for the studied sample was 4.7±3.1. The highest reported stigma category was mild stigma: Social stigma towards current COVID-19 patients (88.2%), Social stigma toward recovered COVID-19 patients (64.2%), Negative self-image for being a COVID-19 patient; perceived self-stigma (71.6%) and total stigma score (88.2%) respectively. The overall stigma score was negatively associated with a higher level of education and getting information from healthcare workers and positively associated with getting information from social networks.

**Conclusion:**

Social and self-stigma related to COVID-19 infection was mild from the Egyptian perspective but found in a large proportion of the population and mainly affected by getting information from healthcare workers or through social media and being more among those with lower education levels. The study recommends more legislative control on social media for disseminating health-related information and conducting awareness campaigns to counteract these adverse effects.

## Introduction

The social stigma associated with diseases has been defined as a negative social judgment that results in refusal and/or rejection. This negative judgment may be directed towards those suffering from diseases or self (self-stigma) [1]. Stigmatization causes, in addition to discrimination, many other harmful effects as concealing disease and delaying access and, consequently, utilization of health care [2].

Over the last decades, social stigma has been associated with various mental disorders and sexually transmitted diseases [3]. However, social stigma recently has spread to pandemic-causing infections with the emergence and re-emergence of several infectious diseases in small outbreaks and even pandemics. Stigma has long been a dominant concern of people with infectious diseases due to fears of contagion and risk of death [4].

There have been outbreaks of Ebola and Zika viruses in the past ten years, in addition to an influenza A (H1N1) in the 2009 pandemic, where an associated stigma was reported [5]. This infectious outbreaks-associated stigma was an obstacle that hindered the adoption of preventive behaviours, which led to more severe consequences, increased virus transmission, and a challenge to the prevention and control efforts to control these outbreaks [6].

Recently, the COVID-19 pandemic has affected the whole world; it is caused by the SARS-CoV-2 virus and has had multiple genetic variants and a myriad of related symptoms in addition to a rising toll of fatality [7]. The fear of COVID-19 infection is a major driver for disease-related discrimination and stigma, which adds to the fears generated from the uncertainties around the disease cause of infection and fatality and perception of guilt as a source of infection [8–10].

The double burden of previous quarantine enforcement by law and both dis/misinformation on social media are also direct causes of stigma [11]; other drivers include a lack of awareness of one’s rights not to be stigmatized and the weak policies against discrimination and the reluctance of the authorities to enforce them [12].

Most research reported experiencing stigmatization in individuals not only with current COVID-19 infection and in those recovered patients and their families, Asian minorities, and healthcare workers (HCWs) who have experienced COVID-19-associated stigma [10, 13–15]. This study aims to assess social and self-stigma related to COVID-19 infection and other associated factors among a sample of Egyptians during the pandemic.

## Materials and methods

### Study design and sample size

A cross-sectional study was conducted in July 2021 among 553 adult Egyptians. A sample size of 334 participants was calculated at a 95% confidence level and an alpha error of 5% using Epi Info 7 software based on the prevalence of stigma among Egyptian healthcare workers (HCWs) who are involved in treating COVID-19 patients as they were the closest population in culture and perceived attitudes to our target population [16].

### Data collection

An online questionnaire was designed following the standard approach suggested in the behavioural insights research for COVID-19 by guidance to the Member States in the WHO European Region [17], the modified Berger HIV stigma scale [18], and after a review of the previously published studies on infectious disease-related stigma [5, 16].

The online questionnaire was distributed through various social networking packages using the snowball sampling method, starting from known individuals, and then they were asked to share the survey with others. The estimated time to fulfil the questionnaire was about 10–15 minutes.

The questionnaire started with an introduction that explained the study’s rationale and objective, explaining that participation is voluntary, ensuring the confidentiality of responses and that completing the questionnaire implied the participants’ consent to participate in the study (S1 Appendix).

The questionnaire was designed in the native language of Egypt; Arabic, and was formed of 3 sections:

- **Sociodemographic questions:** the section included questions about age, gender, education, and residence.
- **Sources of Knowledge regarding COVID-19:** using multiple options formats, which included social networks, the Egyptian Ministry of Health and Population website, Health care workers, Television and radio, posters and brochures, and Newspapers and magazines
- **Questions about COVID-19 infection stigma, formed of 3 sub-scales:** social stigma toward current COVID-19 patients (12 questions), Social stigma toward recovered COVID-19 patients (6 questions) and negative self-image if being a COVID-19 patient (perceived self-stigma) (7 questions). The respondents were then given a three-point Likert scale (agree, not sure, disagree). For each response denoting stigma, a score of one was given, while the responses denoting no stigma scored 0.

The total score was calculated by adding up the responses denoting stigma in each sub-scale (Social stigma toward current COVID-19 patients, social stigma toward recovered COVID-19 patients and Negative self-image if being a COVID-19 patient; self-stigma) and then converting it into a percent-score (by division by the maximum score and then multiplying by 100). Then, using the same method, the three sub-scales were added to calculate the total COVID-19-related stigma score.

The current study followed the methodology of previous studies methods in categorizing stigma due to the lack of universal cut-off points for stigma scores [16, 19–21]. Each sub-scale and the total stigma score were categorized into three categories; mild, moderate, and severe stigma, using the 33rd and 66th percentile cut-off values of the distribution of scores.

### Validity and reliability

The content validity of the questionnaire was evaluated by ten experts of different disciplines: family medicine, public health and tropical medicine, Suez Canal University. After analysing the views of the experts, the authors revised language and questions that were unclear, confusing, or perhaps unacceptable to the participants. The questionnaire was then examined for face validity in a pilot research with 15 participants to ensure the questions’ clarity, appropriateness, and simplicity, as well as their multiple-choice format. Minor modifications of a few questions followed feedback from the pilot study.

Confirmatory factor analysis (CFA) was conducted to assess construct validity. The CFA findings revealed a statistically significant association between the survey items. The Cronbach’s alpha values were used to assess the questionnaire’s internal consistency. Cronbach’s alpha = 0.753 for the full scale was found in the current study’s pilot trial, showing acceptable reliability [22]. However, for the three sub-scales, it was = 0.667, = 0.779, and = 0.642, respectively.

#### Statistical analysis

Data were coded, entered, and analysed using Microsoft Excel version 2016. Data analyses were performed using IBM-SPSS software version 22.0. Sociodemographic characteristics, sources of Knowledge regarding COVID-19 and answers to stigma questions were presented using descriptive statistics, including frequencies and percentages (%). The scores for the stigma total and subscales were presented using mean ±SD.

A Chi-square test was used to test the statistical significance of categorical data. Fisher’s exact test was used whenever Chi-square test assumptions were violated (i.e., when more than 20% of the expected values were less than five or cell values equal 0). Multivariable regression analysis was used to assess factors affecting the overall COVID-19-related stigma score. P-value < 0.05 was considered statistically significant.

#### Ethical considerations

The study was approved by the Research Ethics committee (REC) of the Faculty of Medicine, Suez Canal University, with a code of 4203. The study participants provided electronically written informed consent after being informed about the purpose of the study and the importance of the online form before data collection.

## Results

### Participants’ general characteristics

A total of 553 participants were studied. Most of the participants were within the age range of 18–65 years old. Most of them (n = 399, 72.2%) were females, and with university level of education (n = 349, 63.1%). The most reported Source of Knowledge regarding COVID-19 in order was through social networks (n = 408, 73.8%), the website of the Egyptian Ministry of Health and Population (n = 290, 52.4%) and healthcare workers (n = 284, 51.4%), while the least reported source was Newspapers and magazines (n = 35, 6.3%) (Table 1).

**Table 1:** General characteristics of the studied participants (n = 553).

General characteristics		Frequency	Percent
**Gender**	*Male*	154	27.8
	*Female*	399	72.2
**Education**	*Primary school*	6	1.1
	*Preparatory school*	8	1.4
	*High school*	45	8.1
	*University*	349	63.1
	*Post-graduate*	145	26.2
**Source of information as regarding COVID-19**			
	*Egyptian Ministry of Health and Population website*	290	52.4
	*Health care workers*	284	51.4
	*Television and radio*	213	38.5
	*Posters and brochures*	39	7.1
	*Newspapers and magazines*	35	6.3

### Social stigma toward current COVID-19 patients

As regards social stigma toward current COVID-19 patients, the most reported situations were: not dealing with people coming back from abroad even while observing precautionary measures (n = 143, 25.9%), being afraid of dealing with people who just come back from abroad (n = 127, 23%), not supporting a close friend who has been infected with COVID-19 (n = 104, 18.8%), being afraid of a neighbour, a close relative or friend working in the medical field (n = 77, 13.9%) and victims of COVID-19 should be buried away from usual burring places (n = 68, 12.3%). However, there was no stigmatization directed towards poor and uneducated people. Also, the least reported stigmatizing situations were: COVID-19-infected persons should feel ashamed (n = 12, 2.2%) or be blamed for their illness (n = 3, 0.5%).

### Social stigma toward recovered COVID-19 patients

As regards social stigma toward those currently infected with COVID-19, the most reported s situations where: after recovery, they were not able to usually deal with COVID-19 patients released from quarantine (n = 179, 32.4%) or allow their family members to do so either (n = 245, 44.3%). However, all other situations were reported less than 10%.

### Negative self-image if being a COVID-19 patient

Regarding negative self-image regarding being a COVID-19 patient (self-stigma), the most reported situations where it would be a catastrophic situation if people suspected their infection with COVID-19 (n = 215, 38.9%) they would keep it secret if one of their family members were infected (n = 123, 22.2%), they would also keep it secret if they have infected themselves (n = 99, 17.9%), in addition to feeling guilty if got the infection and blame it on their careless behaviours and not following social distancing (n = 91, 16.5%). They would also keep it secret if one of a family member died due to their infection (n = 69, 12.5%). However, all other situations were reported with less than 10%.

### COVID-19-related stigma score

**Fig 1:**
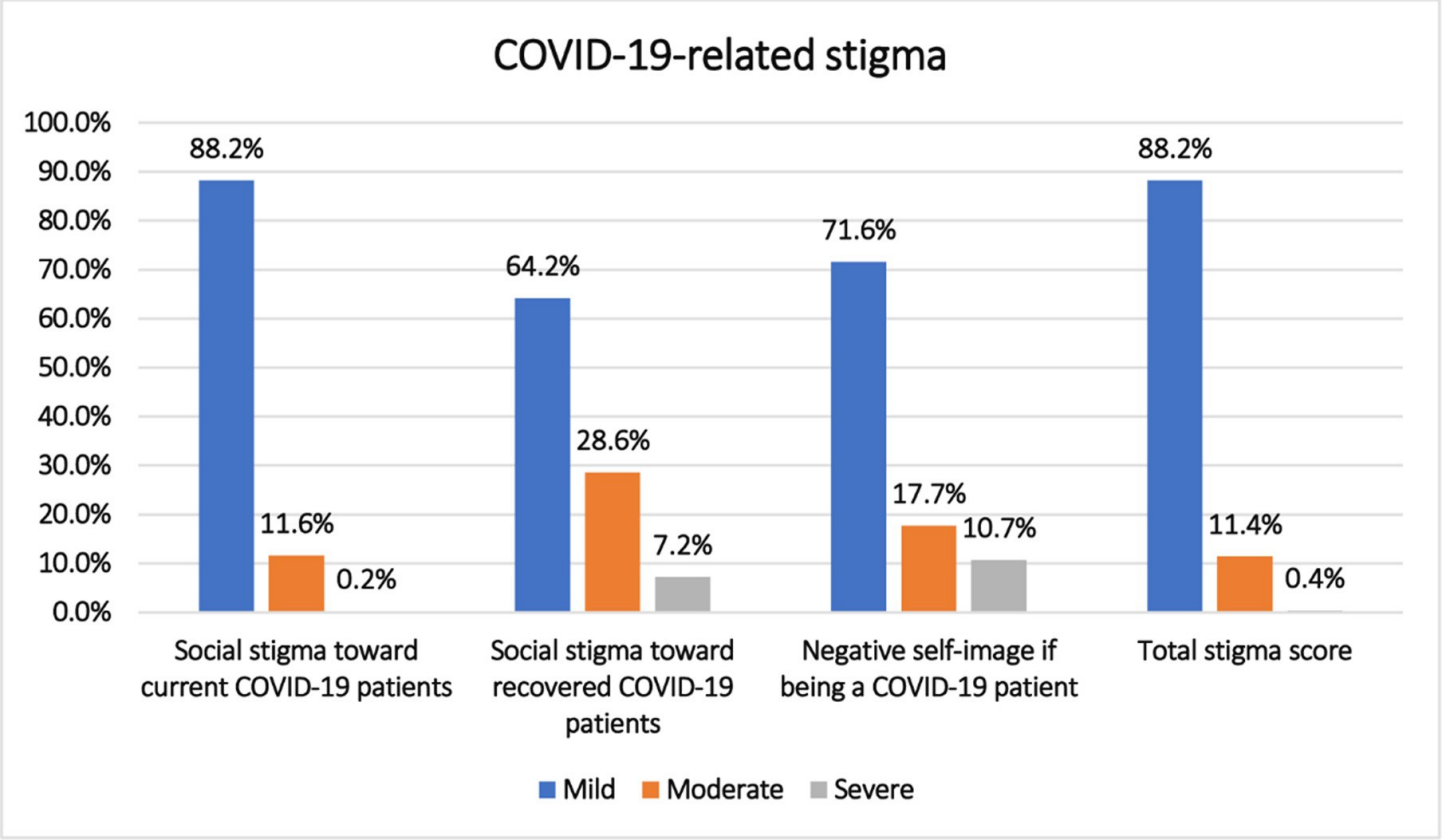
Total COVID-19-related stigma and subscales among the studied participants (n = 553).

The mean overall COVID-19-related stigma score was 4.7±3.1. The mean scores for the subscales were: 1.35±1.6 for social stigma toward current COVID-19 patients, 1.1±1.5 for social stigma toward recovered COVID-19 patients, and 2.1±1.4 for negative self-image if being a COVID-19 patient. The highest reported stigma category, for sub-scales and total, was mild stigma as the following: Social stigma toward current COVID-19 patients (n = 488, 88.2%), Social stigma toward recovered COVID-19 patients (n = 355, 64.2%), negative self-image for being a COVID-19 patient; perceived self-stigma (n = 396, 71.6%) and total stigma score (n = 488, 88.2%) respectively (Fig 1).

The relation between general characteristics and total stigma score was not statistically significant (P-value >0.05) (Table 2). The relation between sources of information and total stigma score showed that the participants getting their information from social networks have a higher total stigma score (P-value <0.05) (Table 3).

**Table 2:** Relation between general characteristics and total stigma categories among the studied participants (n = 553).

General characteristics *Frequency (%)*		Total stigma score			
		Mild	Moderate	Severe	*P-value*
**Gender**	*Male*	135(27.7)	19(30.2)	0(0)	*0*.*822*
	*Female*	353(72.3)	44(69.8)	2(100)	
**Education**	*Primary school*	6(1.2)	0(0)	0(0)	*0*.*283*
	*Preparatory school*	5(1)	3(4.8)	0(0)	
	*High school*	38(7.8)	7(11.1)	0(0)	
	*University*	310(63.5)	38(60.3)	1(50)	
	*Post-graduate*	129(264)	15(23.8)	1(50)	

**Table 3:** Relation between source of information and total stigma categories among the studied participants (n = 553).

Source of information *Frequency (%)*	Total stigma score			
	Mild	Moderate	Severe	*P-value*
**Social networks**	349(71.5)	57(90.5)	2(100)	0.001*
**Egyptian Ministry of Health and Population website**	260(53.3)	29(46)	1(50)	0.644
**Health care workers**	254(52)	29(46)	1(50)	0.711
**Television and radio**	185(37.9)	27(42.9)	1(50)	0.639
**Posters and brochures**	37(7.6)	2(3.2)	0(0)	0.391
**Newspapers and magazines**	33(6.8)	2(3.2)	0(0)	0.483

*P-value is statistically significant.

As regards the multivariable regression models for predicting the total stigma score, participants’ overall COVID-19-related stigma score was negatively associated with higher level of education (β = -0.091, 95% CI: -0.802, -0.037, p = 0.032), positively associated with using social networks for information (β = 1.545, 95% CI: 0.951, 2.139, p = 0.001) and negatively associated with getting information from health care workers (β = 0.271, 95% CI: -1.093, -0.026, p = 0.04). These relations were all statistically significant (P-value <0.05) (Table 4).

**Table 4:** Multivariable regression analysis of factors affecting overall COVID-19-related stigma score (n = 553).

Independent variables	Unstandardized Coefficients		Standardized Coefficients	t	*P-value*	95% Confidence Interval for B	
	B	Std. Error	Beta			Lower Bound	Upper Bound
**Age**	-0.006	0.164	-0.002	-0.037	*0*.*97*	-0.328	0.316
**Gender**	-0.562	0.303	-0.079	-1.854	*0*.*064*	-1.156	0.033
**Residence**	-0.046	0.093	-0.021	-0.492	*0*.*623*	-0.229	0.137
**Education**	-0.42	0.195	-0.091	-2.156	*0*.032*	-0.802	-0.037
**Social networks**	1.545	0.302	0.213	5.107	*0*.001*	0.951	2.139
**Egyptian Ministry of Health and Population website**	-0.453	0.272	-0.071	-1.667	*0*.*096*	-0.988	0.081
**Health care workers**	-0.559	0.271	-0.087	-2.062	*0*.04*	-1.093	-0.026
**Television and radio**	0.334	0.279	0.051	1.196	*0*.*232*	-0.215	0.883
**Posters and brochures**	-0.538	0.531	-0.043	-1.013	*0*.*312*	-1.582	0.506

*P-value is statistically significant.

## Discussion

The results of the current study reported stigmatization of mild degree among more than three-quarters of the studied participants toward current COVID-19 patients (88.2%), about two-thirds toward recovered COVID-19 patients (64.2%), and about three-quarters for negative self-image for being a COVID-19 patient; perceived self-stigma (71.6%) respectively.

This was consistent with what was found by Sangma et al. [10] in their study in India, as they reported a higher proportion of significant social stigma and discrimination in terms of personalized stigma and negative self-image. Patel et al. [15] also in India reported similar results of stigmatization, however, on different populations; of healthcare workers, as well as the findings of Lu et al. [8] among Taiwanese frontline healthcare workers. However, Jayakody et al. [23] in Sri Lanka reported a lower proportion of the population suffering COVID-19-related stigma, despite having severe adverse consequences at the individual and family levels.

This relatively lower degree of stigma but in large proportion scale of the studied population could be explained by the fact that the pandemic, by the time of data collection, was at its second year with widely distributed active cases all over the country [24]. This made the population more oriented, accepting, and contacting active cases almost everywhere.

In Egypt, a recent survey conducted by the Central Agency for Public Mobilization and Statistics (CAPMAS) reflected the effect of the slowdown in economic and business activity, as more than a quarter of respondents stated losing their jobs due to the COVID-19 pandemic sequences. This uprising of financial hardship due to increased unemployment has increased grievances and social stigma [24].

Stigma towards COVID-19 is different from the case of HIV in one significant aspect. COVID-19-related stigma is mainly due to the fear of its ease of transmissibility, high spread, and fatality among the population, while that related to HIV is linked to the unacceptable break of religious boundaries in Egypt, where the main religions are Islam, followed by Christianity [13, 25]. This fact is a possible explanation for the paradoxical finding of a mild degree but a large proportion of COVID-19-related stigma in the current study.

COVID-19-related stigma was negatively associated with a higher level of education in the current study. This association is consistent with the finding of Abdelhafiz et al. [26] study on Knowledge, perceptions, and attitude of Egyptians towards COVID-19, where less educated people thought that infection is associated with stigma as the Yuan et al. [27] systematic review and meta-analysis study.

The higher level of education provides enhanced access to correct and precise information and, as a result, builds a well-established stone of Knowledge about infectious diseases, infectiousness, and preventive measures. This, in turn, facilitates their ability to discriminate between accurate information and misinformation. On the contrary, those with poor Knowledge, as a disadvantage given by the lower level of education, got misled by the factitious news and wrong information [28].

Conversely, COVID-19-related stigma was positively associated with using social networks for information and negatively associated with getting information from healthcare workers in the current study. That finding agreed again with Yuan et al. [27] systematic review and meta-analysis study, and Islam et al. [29] showed that a one-point increase in baseline knowledge is associated with a 0.51-point decrease in baseline stigma index.

Social media provides both an opportunity and a challenge for the public during the COVID-19 pandemic. The opportunity is that it allows information about the disease and prevention but puts them on a great challenge of being affected by misinformation, especially when they are not competent enough to distinguish it, becoming a direct source of fear, anxiety, and stigma [30].

### Limitations

The current study findings should be viewed in light of some limitations. The study’s observational nature was conducted to explore the situation in this new area of inquiry. It was not used to infer causal relationships. And due to the COVID-19 critical situation to achieve social distance, the researchers used the online data collection method. Consequently, the researchers recommend conducting further studies using face-to-face interviews using a probability sampling technique.

## Conclusion

Social and self-stigma related to COVID-19 infection were mild from the Egyptian perspective but found in a large proportion of the population. However, the perceived stigma was mainly affected negatively by the source from where the population got their health-related information. On the other hand, getting this information from healthcare workers better impacts perceiving stigma. Finally, being with lower education levels makes the population more vulnerable to stigmatizing COVID-19 cases.

### Recommendations

Based on the current study’s findings, we recommend more legislative actions for the control of social media regarding disseminating health-related information and to increase awareness campaigns to counteract the adverse effects of getting such information from social media instead of the suitable channels for this.

## Supporting information

S1 Appendix (DOCX)

S1 Data (XLSX)
