# Supplementary material for: Social and self-stigma during COVID-19 pandemic: Egyptians’ perspectives
Source: PLoS One. 2023 Apr 20;18(4):e0284582. doi: 10.1371/journal.pone.0284582 (PMC10118092; doi:10.1371/journal.pone.0284582)
Supplement: S1 Appendix — (DOCX) [file pone.0284582.s001.docx]

**Appendix (1)**

| **Social stigma toward current COVID-19 patients** | **Response** | | |
| --- | --- | --- | --- |
|  | Agree | Not sure | Disagree |
| COVID-19 infected persons should feel ashamed. |  |  |  |
| COVID-19 infected persons should be blamed for their illness. |  |  |  |
| Only uneducated persons get infected with COVID-19. |  |  |  |
| Only poor persons get infected with COVID-19. |  |  |  |
| You would support a close friend of yours has COVID-19. |  |  |  |
| You would be frightened if you have a neighbor, a close relative or friend working in the medical field. |  |  |  |
| You would be frightened if you have to deal with people of Asian origins. |  |  |  |
| You would be frightened if you have to deal with people just coming back from abroad. |  |  |  |
| Even with precautions, you must not to deal with a neighbor, a close relative or friend working in the medical field. |  |  |  |
| Even with precautions, you must not to deal with people of Asian origins. |  |  |  |
| Even with precautions, you must not to deal with people coming back from abroad. |  |  |  |
| Victims of COVID-19 should be buried away from usual burring places. |  |  |  |
| **Social stigma toward recovered COVID-19 patients** |  |  |  |
| After recovery, COVID-19 patients will be able to return completely to their life. |  |  |  |
| After recovery, you would deal easily with COVID-19 patients treated at home. |  |  |  |
| After recovery, you would deal easily with COVID-19 patients released from quarantine. |  |  |  |
| After recovery, you would allow your family members to deal easily with COVID-19 patients treated at home. |  |  |  |
| After recovery, you would allow your family members to deal easily with COVID-19 patients released from quarantine. |  |  |  |
| If you were a Chairman of work, would you give permission to a COVID-19 recovered person to work at your workplace. |  |  |  |
| **Negative self-image if being a COVID-19 patient (perceived self-stigma)** | | | |
| I would feel ashamed if I got COVID-19 infection. |  |  |  |
| I would feel ashamed if one of my family members got COVID-19 infection. |  |  |  |
| I would feel guilty if I got COVID-19 infection because of my careless behaviors as not following social distancing. |  |  |  |
| I would keep it secret if I got COVID-19 infection. |  |  |  |
| I would keep it secret if one of my family members got COVID-19 infection. |  |  |  |
| I would keep it secret if one of my family members died with COVID-19 infection. |  |  |  |
| It would be a catastrophic situation if people suspected I got COVID-19 infection. |  |  |  |
